# Long-term economic evaluation of the recombinant *Mycobacterium tuberculosis* fusion protein (EC) test for the diagnosis of *Mycobacterium tuberculosis* infection

**DOI:** 10.3389/fphar.2023.1161526

**Published:** 2023-05-16

**Authors:** Sha Diao, Zheng Liu, Dan Liu, Xiao Cheng, Linan Zeng, Xue-Feng Jiao, Zhe Chen, Xiaofeng Ni, Siyi He, Bin Wu, Deying Kang, Chaomin Wan, Rongsheng Zhao, Huiqing Wang, Lingli Zhang

**Affiliations:** ^1^ Department of Pharmacy, West China Second University Hospital, Sichuan University, Chengdu, China; ^2^ Evidence-Based Pharmacy Center, West China Second University Hospital, Sichuan University, Chengdu, China; ^3^ NMPA Key Laboratory for Technical Research on Drug Products In Vitro and In Vivo Correlation, Chengdu, China; ^4^ Key Laboratory of Birth Defects and Related Diseases of Women and Children, Sichuan University, Ministry of Education, Chengdu, China; ^5^ West China School of Medicine, Sichuan University, Chengdu, China; ^6^ West China School of Pharmacy, Sichuan University, Chengdu, China; ^7^ Department of Pharmacy, Renji Hospital Affiliated with the School of Medicine, Shanghai Jiaotong University, Shanghai, China; ^8^ Chinese Evidence-Based Medicine Center, West China Hospital, Sichuan University, Chengdu, China; ^9^ Department of Pediatrics, West China Second University Hospital, Sichuan University, Chengdu, China; ^10^ Department of Pharmacy, Peking University Third Hospital, Beijing, China; ^11^ Medical Simulation Centre, West China Second University Hospital, Sichuan University, Chengdu, China

**Keywords:** recombinant *Mycobacterium tuberculosis* fusion protein (EC), purified protein derivative of tuberculin (TB-PPD), *Mycobacterium tuberculosis* infection, decision tree-markov model, cost-utility

## Abstract

**Background:** Tuberculosis continues to be a significant global burden. Purified protein derivative of tuberculin (TB-PPD) is one type of tuberculin skin test (TST) and is used commonly for the auxiliary diagnosis of tuberculosis. The recombinant *Mycobacterium tuberculosis* fusion protein (EC) test is a new test developed in China.

**Objective:** Evaluate the long-term economic implications of using the EC test compared with the TB-PPD test to provide a reference for clinical decision-making.

**Methods:** The target population was people at a high risk persons of being infected with *Mycobacterium tuberculosis*. The outcome indicator was quality-adjusted life years (QALY). A cost–utility analysis was used to evaluate the long-term economic implications of using the EC test compared with the TB-PPD test. We employed a decision tree–Markov model from the perspective of the whole society within 77 years.

**Results:** Compared with the TB-PPD test, the EC test had a lower cost but higher QALY. The incremental cost–utility ratio was −119,800.7381 CNY/QALY. That is, for each additional QALY, the EC test could save 119,800.7381 CNY: the EC test was more economical than the TB-PPD test.

**Conclusion:** Compared with the TB-PPD test, the EC test would be more economical in the long term for the diagnosis of *M. tuberculosis* infection according our study.

## Introduction

Tuberculosis is a chronic infectious disease caused by *Mycobacterium tuberculosis* (MTB) infection. According to estimates published by the World Health Organization (WHO), tuberculosis is the 13th leading cause of death worldwide and the number-one cause of death from a single infectious agent. In 2020, it was anticipated that tuberculosis will rank as the second leading cause of death from a single infectious agent, after Coronavirus disease-2019 (GBD 2019 Diseases and Injuries Collaborators, 2020; World Health Organization, 2021). According to the *Global Tuberculosis Report 2021* published by the WHO, nearly one-third of the worldwide population is infected with MTB, with ∼2 billion infected people, ∼9.9 million new patients with tuberculosis, and ∼1.514 million deaths from tuberculosis. In China, the number of MTB infections is ∼350 million, and there are 842,000 new patients with tuberculosis, of which ∼32,000 people will die of tuberculosis (Cui et al., 2020; World Health Organization, 2020; World Health Organization, 2021). If people are infected with MTB, 95% will have latent tuberculosis infection (LTBI), and there will be a 5%–10% probability of developing into active tuberculosis (ATB) in their lifetime. Once they have ATB, they will become a new source of tuberculosis infection (He et al., 2018; World Health Organization, 2019).

To eliminate tuberculosis, early identification of LTBI and providing preventive treatment are required (World Health Organization, 2015; Chinese Center for Disease Control and Prevention, 2021). LTBI does not carry the corresponding clinical symptoms, and evidence cannot be provided by imaging or bacteriological tests, so it can be diagnosed only by immunological methods (Zhou et al., 2021).

Purified protein derivative of tuberculin (TB-PPD) is a type of tuberculin skin test (TST). The TB-PPD test is employed commonly for diagnosing of LTBI in clinical practice. The criteria for a positive result is as follows: 1) Average diameter (sum of transverse and longitudinal diameters, divided by 2) of induration ≥6 mm 48–72 h later; 2) Blister, necrosis (skin breakdown) or lymphadenitis are interpreted as strong positive reactions. However, TB-PPD has many identical or similar antigenic components with those in Bacille Calmette-Guérin (BCG) vaccine and non-tuberculous mycobacteria (Pai et al., 2014; Ma et al., 2022).

Testing using recombinant *Mycobacterium tuberculosis* fusion protein (EC) was approved for marketing by China in 2020. EC is made from recombinant-EC obtained after fermentation, isolation and purification of *Escherichia coli* showing high expression of the specific ESAT6-CFP10 gene of MTB. The criteria for a positive result is as follows: 1) Average diameter (sum of transverse and longitudinal diameters, divided by 2) of redness or induration ≥5 mm 48–72 h later; 2) Blister, necrosis (skin breakdown) or lymphadenitis are interpreted as strong positive reactions.

Here, we constructed a decision tree–Markov model and used a cost–utility analysis to evaluate the long-term economic implications of using the EC test compared with using the TB-PPD test within 77 years. In this way, we aimed to provide a reference for clinical decision-making.

## Methods

### Model structure

The target population was high-risk persons with MTB infection: close contacts of people with etiologically positive pulmonary tuberculosis; individuals infected with the human immunodeficiency virus (HIV); people receiving immunosuppressive treatment or other immunocompromised people (Pai et al., 2014; Chinese Center for Disease Control and Prevention, 2021; Zhou et al., 2021; Ma et al., 2022).

The outcome indicator was quality-adjusted life years (QALY). The cost–utility analysis was used with a decision tree–Markov model from the perspective of the whole society. EC was 0.3 mL/bottle. TB-PPD was 1 mL:50 IU/bottle. In our model, the duration of conventional anti-tuberculosis treatment was from 6 months to 12 months. The duration of preventive treatment was from 3 months to 9 months. Therefore, the model took 1 year as one cycle. People of all ages are susceptible to tuberculosis, so the starting age of the model was set to 0 years, and the end of the cycle was set to 77 years (average life expectancy in China).

**FIGURE 1:**
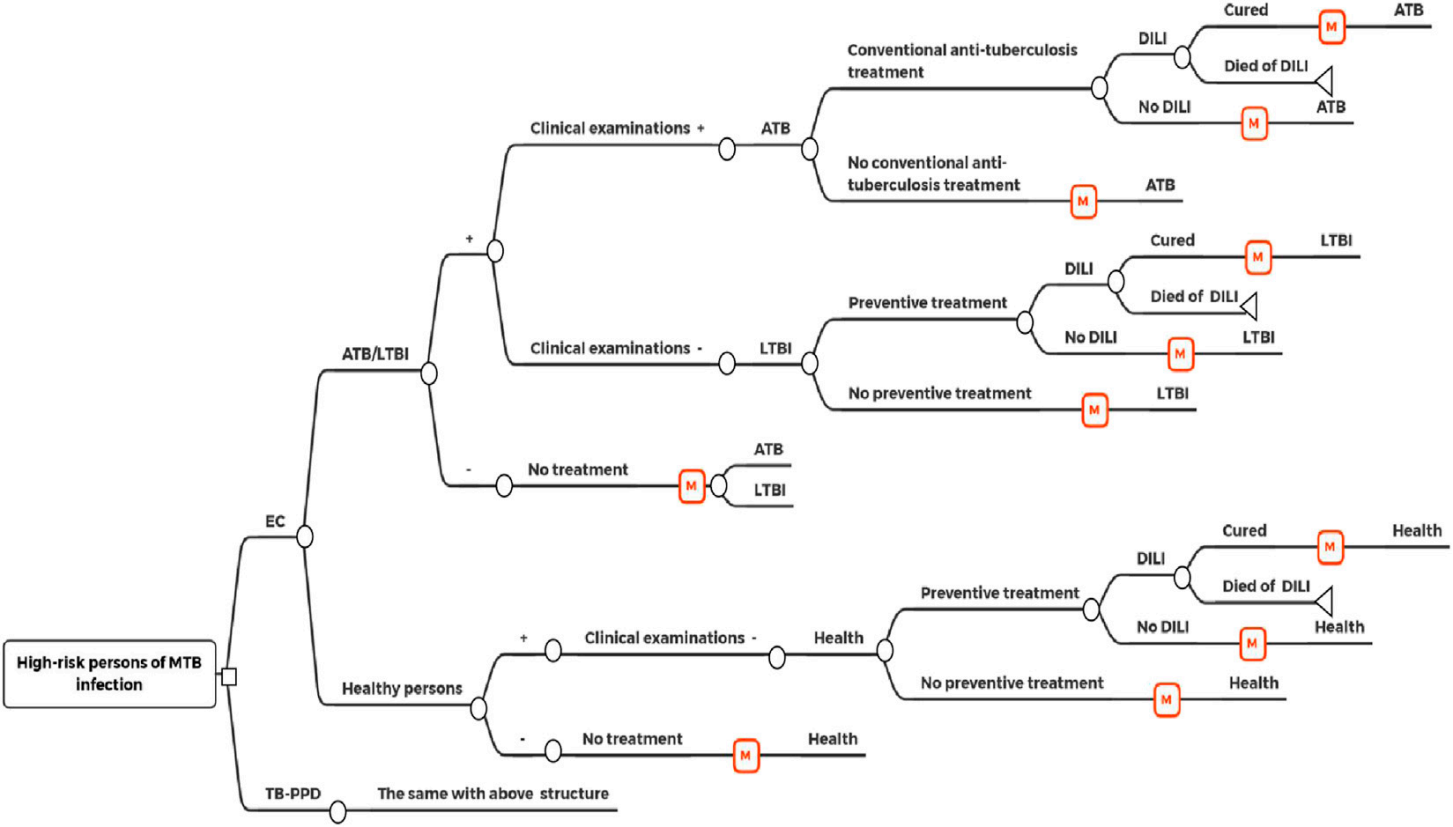
Decision tree-markov model.

**FIGURE 2:**
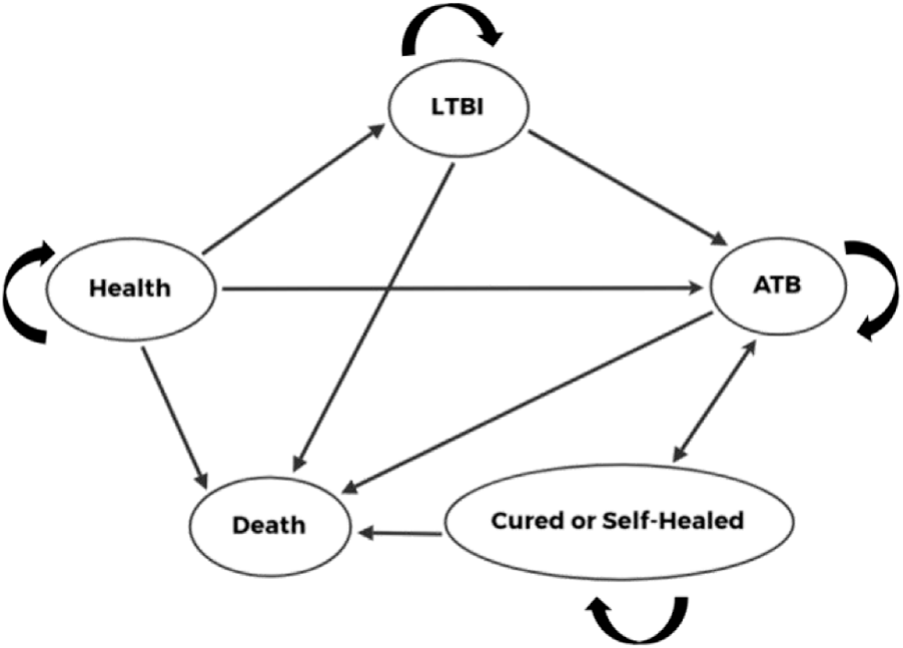
Markov states.

MTB: *Mycobacterium tuberculosis*; EC: Recombinant *Mycobacterium tuberculosis* fusion protein (EC); TB-PPD: Purified protein derivative of tuberculin (TB-PPD); ATB: Active tuberculosis; LTBI: Latent tuberculosis infection; DILI: Drug-induced liver injury.

The disease were divided into five Markov states: “health”, “LTBI”, “ATB”, “cured or self-healed” and “death”. In our model, the target population would receive an EC test or TB-PPD skin test, respectively. If the result was negative, then they would not receive clinical treatment. If the result was positive, then they would be diagnosed as having ATB or LTBI through further clinical examinations (medical history, imaging, etiology). Patients diagnosed with ATB would receive conventional anti-tuberculosis treatment. Patients diagnosed with LTBI would receive preventive treatment. All patients receiving treatment had the potential to develop drug-induced liver injury (DILI). The target population was entered into the Markov model based on different states, and was cycled according to the transition probability between states (Figure 1; Figure 2).

### Model assumptions

The target population was vaccinated with BCG. All study participants complied with treatment. Each person could be in only one state, and undergo state transition only after treatment cessation. The probability of each event occurring in patients during the cycle remained unchanged.

### Model parameters

The parameters of our model were: branch probabilities; transition probabilities between each Markov state; cost value; utility value. Branch probabilities comprised the: sensitivity and specificity of the EC test and TB-PDD test; prevalence of ATB and LTBI; proportion of participants receiving conventional treatment and preventive treatment; prevalence of and mortality due to DILI.

Costs included the: cost of the EC test or TB-PDD test; cost of clinical examination; cost of DILI treatment; treatment-related cost of ATB or LTBI. The treatment-related cost included: direct medical cost (cost of outpatient visits, hospitalization, self-purchased drugs); direct non-medical cost (cost of travel and meals for patients and their families); indirect cost (wage loss of patients and their families due to illness). Utility values were measured by QALY. The discount rate was also included.

The average level of the whole age group for each parameter was taken as a model parameter. The latest research based on a Chinese population was preferred. If there were different values for the same parameter in multiple studies, the weighted average was calculated as the baseline value, and the upper limit and lower limit among all studies were taken as the range of the parameter. If the range could not be obtained, the upper limit and lower limit were estimated based on ± 5% of the baseline value. For parameters that could not be obtained, studies based on non-Chinese populations or expert consultation were used.

### Cost–utility analysis

The incremental cost–utility ratio (ICUR) was calculated based on our model. The willing-to-pay (WTP) threshold was equal to 1–3-times the gross domestic product (GDP) *per capita* (GDP *per capita* of China in 2021 was 80,976 CNY). If ICUR < 1-time GDP *per capita*, then the increased costs were worthwhile, so the model was very economical. If 1-time GDP *per capita* < ICUR < 3-times GDP *per capita*, then the increased costs were acceptable, so the model was economical. If ICUR >3-times GDP *per capita*, then the increased costs were not worthwhile, so the model was not economical.

### Sensitivity analysis

Univariate sensitivity analysis and probabilistic sensitivity analysis were undertaken by varying the values of the parameters mentioned above, and we assessed the impact on the ICUR.

## Results

### Parameters

The values of model parameters are shown in Table 1.

**TABLE 1:** Values of parameters

Name of parameter	Baseline value	Range
Sensitivity of EC	0.9064	0.8750-0.9190
Specificity of EC	0.9272	0.8808-0.9736
Sensitivity of TB-PPD	0.9090	0.8860-0.9280
Specificity of TB-PPD	0.2658	0.2525-0.2791
Prevalence of ATB Wang et al. (2012)	0.0046	0.0043-0.0048
Prevalence of LTBI Gao et al. (2015); Gao et al. (2022); Ma et al. (2022)	0.1881	0.1373-0.2242
Proportion of patients treated for ATB Wang et al. (2012); Li D et al. (2021); Gilmour et al. (2022)	0.9290	0.8190-0.9824
Proportion of patients treated for LTBI Zu (2020a); Zu (2020b); Ren (2020)	0.7130	0.6390-0.8631
Incidence of DILI for conventional treatment Sun et al. (2016); Chinese Medical Association (2019); Zhao et al. (2022)	0.0950	0.0380-0.1290
Incidence of DILI for preventive treatment Pease et al. (2018)	0.0398	0.0100-0.0680
Mortality of DILI Zhao et al. (2020); Huang et al. (2021)	0.0024	0.0024-0.0714
Transition probability from LTBI to ATB with treatment Gao et al. (2018); Xin et al. (2021)	0.0078	0.0003-0.0126
Fatality rate of LTB with treatment Ren (2020)	0.0001	0.0000-0.0002
Cure rate of ATB with treatment The World Bank (2020); Li X et al. (2021); Ruan et al. (2022)	0.9452	0.5710-0.9660
Fatality rate of ATB with treatment Alene et al. (2017); Gilmour et al. (2022)	0.0046	0.0046-0.0264
Recurrence rate of ATB with treatment Shen et al. (2017); Vega et al. (2021); Ruan et al. (2022)	0.0490	0.0226-0.0755
Transition probability from LTBI to ATB without treatment Gao et al. (2017); Gao et al. (2018)	0.0158	0.0058-0.0200
Fatality rate of LTBI without treatment China Statistical Yearbook (2021)	0.0707	0.0707-0.0718
Self-healing rate of ATB without treatment Zu (2020a); Li X et al. (2021)	0.0100	0.0100-0.2500
Fatality rate of ATB without treatment World Health Organization (2021)	0.0400	0.0300-0.0500
Recurrence rate of ATB without treatment Shen et al. (2017)	0.1209	0.1209-0.2340
Incidence of LTBI Gao et al. (2016)	0.0150	0.0150-0.0310
Incidence of ATB World Health Organization (2021)	0.0006	0.0005-0.0007
Natural mortality China Statistical Yearbook. (2021)	0.0707	0.0707-0.0718
Cost of EC	98.00	68.60-98.00
Cost of TB-PPD	136.78	67.80-158.00
Cost of clinical examination Chen et al. (2011); Zu (2020a)	178.93	125.28-232.57
Cost of DILI treatment Chen et al. (2011); Zu. (2020b)	219.62	124.05-240.50
Treatment-related cost of LTBI Zu (2020a)	2158.05	1426.96-2889.14
Treatment-related cost of ATB Zu (2020b)	21112.00	10556.00-63336.00
QALY of LTBI Zu (2020a)	0.9700	0.9500-1.0000
QALY of ATB Zu (2020b)	0.8200	0.6200-0.9300
QALY after ATB cured or self-healed Zu (2020a)	0.9400	0.8700-1.0000
QALY of DILI Dobler et al. (2015)	0.6670	0.4000-0.8000

### Cost–utility analysis

The total cost of the EC test was 7,607.5323 CNY. The total cost of the TB-PPD test was 15,430.5205 CNY. QALY in the EC test was 9.4645. QALY in the TB-PPD test was 9.3992. Compared with the TB-PPD test, the EC test had a lower cost but higher QALY. The ICUR was −119,800.7381 CNY/QALY. That is, for each additional QALY, the EC test could save 119,800.7381 CNY. The EC test was more economical than the TB-PPD test.

### Sensitivity analysis

**FIGURE 3:**
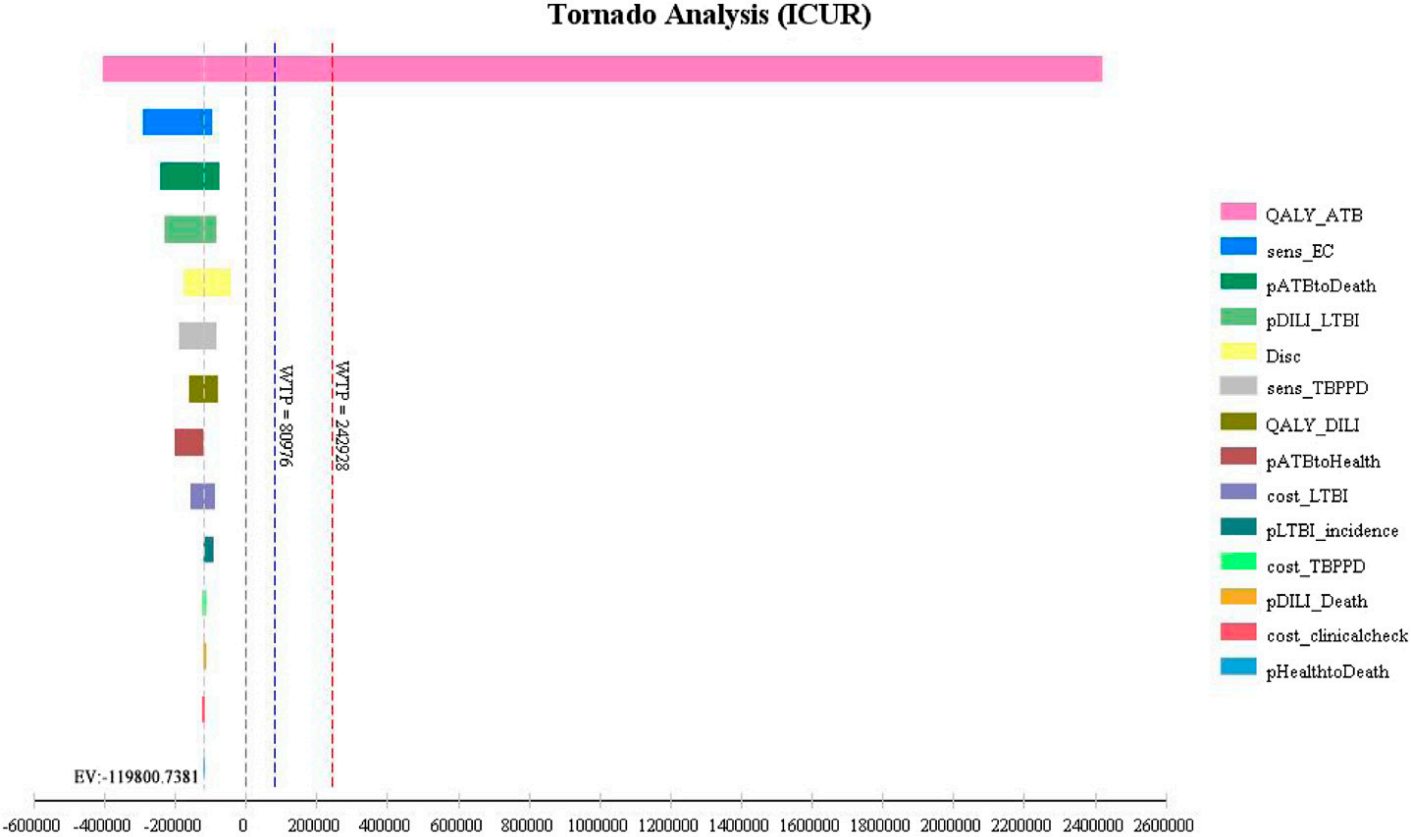
Tornado chart of univariate sensitivity analysis.

Univariate sensitivity analysis showed that the three parameters with the greatest impact on the result were: QALY of ATB; sensitivity of the EC test; fatality prevalence of ATB without treatment. The result was robust if these parameters fluctuated within the range (Figure 3).

QALY_ATB: QALY of ATB; sens_EC: Sensitivity of EC; pATBtoDeath: Fatality rate of ATB without treatment; pDILI_LTBI: Incidence of DILI for preventive treatment; Disc; discount rate; sens_TBPPD: Sensitivity of TB-PPD; QALY_DILI: QALY of DILI; pATBtoHealth: Recurrence rate of ATB with treatment; cost_LTBI: Treatment-related cost of LTBI; pLTBI_incidence: Incidence of LTBI; cost_TBPPD: Cost of TB-PPD; pDILI_Death:Mortality of DILI; cost_clinicalcheck: Cost of clinical examination; pHealthtoDeath: Natural mortality.

**FIGURE 4:**
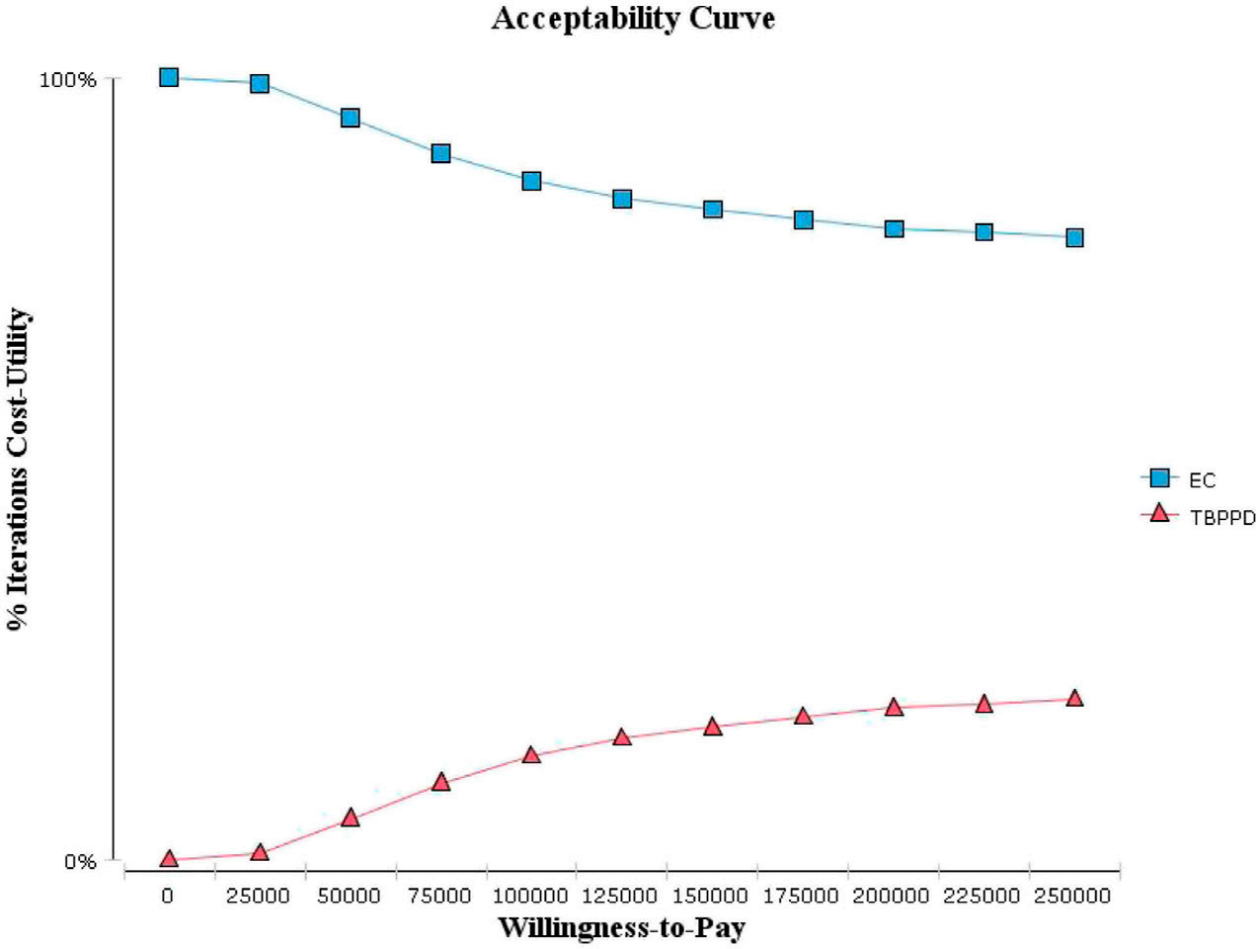
Acceptability curve of cost-utility analysis.

**FIGURE 5:**
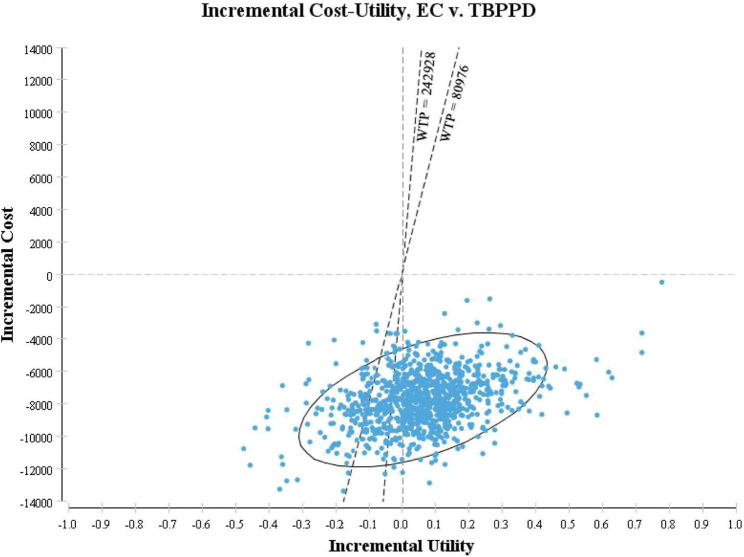
Scatter plot of cost-utility analysis.

EC: Recombinant *Mycobacterium tuberculosis* fusion protein (EC); TBPPD: Purified protein derivative of tuberculin (TB-PPD).

Probabilistic sensitivity analysis showed that the acceptable probability of the EC test was always higher than that of TB-PPD test within the WTP threshold range (Figure 4). The probability of being economical in the EC test was 82.20% if WTP was equal to GDP *per capita*, but 92.80% if WTP was equal to 3-times GDP *per capita* (Figure 5).

## Discussion

Tuberculosis is a major public-health problem worldwide. Early identification of patients suffering from tuberculosis and LTBI detection are the most important measures for prevention and control (Chinese Center for Disease Control and Prevention, 2021). The EC test is a new skin test for detection of MTB infection developed in China. The EC test has been shown to have higher specificity and to be able to distinguish MTB infection from BCG vaccination or other non-MTB infections effectively compared with that using the TB-PPD test (Zhou et al., 2021).

We evaluated the economic viability of using the EC test compared with using the TB-PPD test within the long-term (77 years). We discovered that the EC test was more economical for the diagnosis of MTB infection and subsequent treatment than the TB-PPD test according our study.

A “gold standard” for the diagnosis of LTBI is lacking. Hence, the diagnosis of LTBI can be made only by immunological detection methods such as the EC test or TB-PPD test. The accuracy of the detection method has a crucial and direct influence on the treatment paths of patients. Patients whose diagnosis has been missed will carry a poor prognosis due to a lack of appropriate examination and timely treatment. Healthy people who are misdiagnosed would have a higher economic burden and lower QALY compared with healthy people because they will have received inappropriate examination and treatment. The sensitivity of the EC test was similar to that of the TB-PPD test, but the EC test had higher specificity. Fewer people would be misdiagnosed using the EC test, and they would have lower costs and higher QALY, so the EC test is more economical.

According to the univariate sensitivity analysis, QALY of ATB, the sensitivity of the EC test, and the prevalence of fatality of ATB without treatment had the most prominent impact on the results. The result fluctuated greatly within the range of QALY of ATB, but the conclusion was consistent. If QALY of ATB ≤ 0.65, then the ICUR >3-times GDP *per capita*. This result meant that the TB-PPD test had higher costs and higher QALY compared with the EC test, but the increased costs were not worthwhile. If QALY of ATB >0.65, then the ICUR < 0. This result meant that the EC test had lower costs but higher QALY compared with the TB-PPD test. According to the probabilistic sensitivity analysis, the probability of the EC test being economical increased with increasing WTP thresholds. In summary, the result of the cost–utility analysis was robust.

The EC test was approved for marketing by China in 2020, but economic-evaluation studies related to the EC test are scarce. The WHO (World Health Organization, 2022) conducted a rapid evaluation in 2022 to compare the efficacy, safety and economy of three newer MTB antigen-based skin tests (TBSTs) compared with traditional TSTs and interferon-gamma release assays (IGRAs). The three TBSTs were C-Tb (Serum Institute of India, Pune, India), C-TST (known formerly as the ESAT6-CFP10 test; Anhui Zhifei Longcom, Anhui, China) and Diaskintest (Generium, Moscow, Russian Federation). The C-TST in China is EC mentioned in our study. TBSTs were more accurate and more economical compared with TSTs and IGRAs, though the safety was consistent with that of TSTs. Also, the WHO mentioned that economic evaluation of the EC test was insufficient. Steffen and others (Steffen et al., 2020) compared the cost-effectiveness of Diaskintest, EC test, TB-PPD test and QuantiFERON-TB Gold Plus (QFT-Plus) for the diagnosis of MTB in Brazilian HIV-infected patients by constructing a Markov model: Diaskintest was more economical than other methods. Diaskintest is a new skin test with the same methodology as the EC test developed in 2009 in Russia, but it has not been approved for marketing in China. Steffen and others (Steffen et al., 2020) showed that the EC test had the same effect as that of Diaskintest but had a higher cost. However, the cost of the EC test in China is lower, and we found it to be more economical.

## Limitation and future research

Our study had two main limitations. First, the model parameters were taken from the average level of all age groups in China, so the results may not be applicable to a specific group. Second, the economic evaluation was conducted based only on a model because real-world studies are lacking. Therefore, carrying out an economic evaluation with a prospective study simultaneously would be the best option.

## Conclusion

Compared with the TB-PPD test, the EC test would be more economical in the long term for the diagnosis of MTB infection according our study.

## Data Availability

The original contributions presented in the study are included in the article/supplementary material, further inquiries can be directed to the corresponding authors.
